# Automated ROI-Based Labeling for Multi-Voxel Magnetic Resonance Spectroscopy Data Using FreeSurfer

**DOI:** 10.3389/fnmol.2019.00028

**Published:** 2019-02-14

**Authors:** Benjamin Spurny, Eva Heckova, Rene Seiger, Philipp Moser, Manfred Klöbl, Thomas Vanicek, Marie Spies, Wolfgang Bogner, Rupert Lanzenberger

**Affiliations:** ^1^Department of Psychiatry and Psychotherapy, Medical University of Vienna, Vienna, Austria; ^2^Department of Biomedical Imaging and Image-Guided Therapy, High Field MR Centre, Medical University of Vienna, Vienna, Austria

**Keywords:** MRS, GABA, glutamate, automated labeling, multi-voxel, FreeSurfer

## Abstract

**Purpose**: Advanced analysis methods for multi-voxel magnetic resonance spectroscopy (MRS) are crucial for neurotransmitter quantification, especially for neurotransmitters showing different distributions across tissue types. So far, only a handful of studies have used region of interest (ROI)-based labeling approaches for multi-voxel MRS data. Hence, this study aims to provide an automated ROI-based labeling tool for 3D-multi-voxel MRS data.

**Methods**: MRS data, for automated ROI-based labeling, was acquired in two different spatial resolutions using a spiral-encoded, LASER-localized 3D-MRS imaging sequence with and without MEGA-editing. To calculate the mean metabolite distribution within selected ROIs, masks of individual brain regions were extracted from structural T_1_-weighted images using FreeSurfer. For reliability testing of automated labeling a comparison to manual labeling and single voxel selection approaches was performed for six different subcortical regions.

**Results**: Automated ROI-based labeling showed high consistency [intra-class correlation coefficient (ICC) > 0.8] for all regions compared to manual labeling. Higher variation was shown when selected voxels, chosen from a multi-voxel grid, uncorrected for voxel composition, were compared to labeling methods using spatial averaging based on anatomical features within gray matter (GM) volumes.

**Conclusion**: We provide an automated ROI-based analysis approach for various types of 3D-multi-voxel MRS data, which dramatically reduces hands-on time compared to manual labeling without any possible inter-rater bias.

## Introduction

Magnetic resonance spectroscopy (MRS) enables the quantification of neurotransmitters and several other metabolites in the human brain. Especially glutamate (Glu) and gamma-aminobutyric acid (GABA), which are the main excitatory and inhibitory neurotransmitters within the central nervous system, respectively, have been the focus of neurological (Agarwal and Renshaw, 2012) and psychiatric research (Sanacora et al., 2004; Ramadan et al., 2013; Poels et al., 2014). Both neurotransmitters appear in relatively low concentrations of 5–15 mM for Glu and 1–2 mM for GABA in the human brain (De Graaf, 2007; Haga et al., 2009). Hence, an adequate analysis method is essential for the detection of possible changes. Large voxels, used both in single voxel and multi-voxel MRS, inevitably contain different tissue types, including gray matter (GM), white matter (WM) or cerebrospinal fluid (CSF). Moreover, different tissue types contain varying concentrations of metabolites, which has been reported for GM and WM (Jensen et al., 2005; Bhattacharyya et al., 2011; Zhu et al., 2011; Harris et al., 2015). Furthermore, aberrant neurotransmitter levels within one region, or spatial differences across various regions can have a substantial impact on quantification methods (Gasparovic et al., 2006; Bhattacharyya et al., 2011; Geramita et al., 2011; Gussew et al., 2012). Hence, an adequate analysis approach is crucial for metabolites of high spatial or tissue specific variability.

Up to now most studies used single-voxel approaches with relatively big voxel sizes ranging from 1 to 8 cm^3^ for glutamate and 8–27 cm^3^ for GABA quantification to maximize the signal-to-noise ratio (SNR) in reasonable scan times. Different analysis approaches for single-voxel MRS have been developed thus far. On a basic level MRS data is corrected for the fraction of either solely the CSF (Foerster et al., 2013) or GM (Stagg et al., 2009) in a voxel, which reduces inter-subject variability. Voxel tissue compositions are usually determined by segmentation of additionally acquired T_1_-weighted images using statistical parametric mapping (Ashburner and Friston, 2005) or FMRIB software library (Zhang et al., 2001). In more advanced analysis approaches, composition parameters of GM, WM, or CSF, based on different relaxation times or visibility of water, can be included in a final correction model (Harris et al., 2015; Long et al., 2015; Mikkelsen et al., 2016; Porges et al., 2017).

During the last years an increasing trend towards multi-voxel MRS sequences, both 2D and 3D, has developed to cover larger parts of the brain. This makes more sophisticated data analysis approaches necessary. One simple way of dealing with multi-voxel data is to select single voxels from a multi-voxel grid (Lai et al., 2018). In other approaches, similar to single-voxel data, the volume of interest (VOI) is segmented into GM, WM and CSF and proportions are used as covariates in a final model (Bradley et al., 2016). Depending on the favored metabolites to be analyzed and the field strengths of the scanner voxel sizes can differ tremendously between acquisition methods to obtain a sufficient SNR. While there are approaches to measure small voxels with low SNR, that are clustered for the analysis, others rely on bigger voxels with sufficient SNR, within each voxel, which can either be moved in the grid during the postprocessing (i.e., voxel shifting) to cover regions of interest (ROIs) or using interpolation methods to refine the acquired grid.

Whenever it comes to the definition of ROIs for data analysis, one has to be careful to consider their size and position in the VOI. Several studies use interpolation and manual delineation methods to localize ROIs in multi-voxel data (Mathew et al., 2009; Shungu et al., 2012; Bradley et al., 2016). However, manual masking methods can easily be impaired by systematic errors and suffer from potential inter-rater variability. A first automated ROI-based approach using the metabolite imaging and data analysis system (MIDAS) software and the automated anatomical labeling atlas in the Montreal neurological institute space (Maudsley et al., 2006, 2009; Sabati et al., 2015) was recently developed. However, this approach requires spectral data derived from a specific EPSI sequence to match input criteria, which is not applicable for other MRS sequences and results in difficulties regarding low resolution MRS data.

Hence, this work aims to introduce an automated ROI-based labeling method for multi-voxel MRS data using FreeSurfer. FreeSurfer is a well-established segmentation software allowing for both cortical and subcortical segmentation in the individual space (Fischl et al., 2002; Desikan et al., 2006; Destrieux et al., 2010). FreeSurfer has shown solid results both in healthy as well as atrophic subjects for segmentation purposes (Liem et al., 2015) or cortical thickness evaluations (Seiger et al., 2018). The provided method, which is applicable for different kinds of multi-voxel MRS data sets, aims to reduce inter-rater variability and hands-on time for manual labeling approaches. To investigate the reliability of the proposed labeling method, a comparison with manual labeling and selected voxels of the multi-voxel grid was conducted.

## Materials and Methods

### Magnetic Resonance Imaging

MRS measurements were performed on a 3 Tesla MR Scanner (MAGNETOM Prisma, Siemens Medical, Erlangen, Germany) using a 64-channel head coil at the Medical University of Vienna. This study was approved by the ethical committee of the Medical University of Vienna. Participants gave written consent to participate in this study.

#### Structural Images

For accurate placement of the VOI and further automated segmentation, 3D T_1_-weighted anatomical reference images were acquired *via* a magnetization-prepared rapid gradient-echo (MPRAGE) sequence (TR = 1,800 ms, TE = 2.37 ms, 208 slices, 288 × 288 matrix size, slice thickness = 0.85 mm, voxel size = 1.15 × 1.15 × 0.85 mm^3^, flip angle = 8°, anterior-posterior phase encoding) with a total scan time of 3:39 min.

#### Magnetic Resonance Spectroscopy

##### Low-Resolution GABA-Edited MRS

For spectroscopic measurements, a constant-density, spiral-encoded, 3D-MRS imaging sequence with MEGA-LASER editing, as described in Bogner et al. (2014a) was used. Real-time correction for rigid motion bias (i.e., translations and rotations) and correction of center frequency changes was applied (Bogner et al., 2014a,b). All MRS slices were placed parallel to the anterior commissure–posterior commissure line. VOI was centered to the medial to posterior part of the corpus callosum and to cover the hippocampus bilaterally, with VOI = 80 (l-r) × 90 (a-p) × 80 (s-i) mm^3^ and field of view (FOV) = 160 × 160 × 160 mm^3^. The acquired matrix size of 10 × 10 × 10 (i.e., ~4 cm^3^ nominal voxel size) was interpolated to a 16 × 16 × 16 matrix (i.e., ~1 cm^3^ nominal voxel size) during spectral processing steps. Gradient-echo imaging based shimming with subsequent manual optimization was performed. During the EDIT-ON acquisition, the MEGA-editing pulses (60 Hz Gaussian pulses of 14.8 ms duration) were set to 1.9 ppm, editing the coupled 4CH_2_ triplet of GABA resonating at 3.02 ppm (Andronesi et al., 2010; Mullins et al., 2014). VOI selection *via* LASER and low-power and wide-bandwidth GOIA pulses enabled MEGA editing with an echo time of 68 ms (Bogner et al., 2014a). For real-time correction, volumetric, dual-contrast, echo planar imaging based navigators that update center frequency and head position changes for each pair of EDIT-ON/OFF acquisitions were used (i.e., with a repetition time of 1.6 s, an update occurs every 3.2 s). For 3D-MRSI, 32 acquisition weighted averages and two-step phase cycling were employed, in a total scan time of 15:09 min.

##### High-Resolution Non-edited MRS

Additionally, a short echo time version of the described sequence without spectral editing was used to validate the provided labeling method in MRSI data with higher resolution. Due to insufficient SNR of GABA+ and Glx in reasonable scan times in small voxel sizes a non-edited version of the described sequence was used. The VOI was centered to cover the putamen and pallidum bilaterally, with VOI = 80 (l-r) × 90 (a-p) × 80 (s-i) mm^3^, FOV = 160 × 160 × 160 mm^3^ and an acquired matrix size of 23 × 23 × 12 (i.e., ~0.65 cm^3^ voxel size). To maximize SNR for derived metabolites a TE of 30 ms was used. Shimming procedure and real-time motion correction were conducted as described above. For high-resolution MRSI, 12 acquisition weighted averages and two-step phase cycling were employed, in a total scan time of 17:49 min.

### MRS Data Analysis

All spectra within the VOI were processed automatically with an in-house-developed software tool using MATLAB (R2013a, MathWorks, Natick, MA, USA), Bash (version 4.2.25, Free Software Foundation, Boston, MA, USA) and MINC (MINC Tools, Version 2.0; McConnell Brain Imaging Center, Montreal, QC, Canada), which features a graphical user interface for automatic data processing and employs LCModel software (Version 6.3–1, S. Provencher, LCModel, Oakville, ON, Canada). Three different simulated basis sets were created using GAMMA, one for the EDIT-OFF (containing 21 brain metabolites), one for the difference spectrum [containing GABA+, a combination of glutamate and glutamine (Glx), and total N-acetylaspartate (tNAA)] and one for the non-edited spectra derived from the high-resolution MRS [containing tNAA and total creatine (tCr); (Hnilicová et al., 2016)]. Cramér–Rao lower bounds (CRLB) thresholds were set at 30%. GABA+ and Glx ratios relative to tNAA (GABA+/tNAA and Glx/tNAA) were calculated and tNAA ratios, derived from the high-resolution MRSI were calculated relative to tCr (tNAA/tCr).

### Automated Segmentation and ROI-Based Analysis of Spectral Data

**Figure 1:**
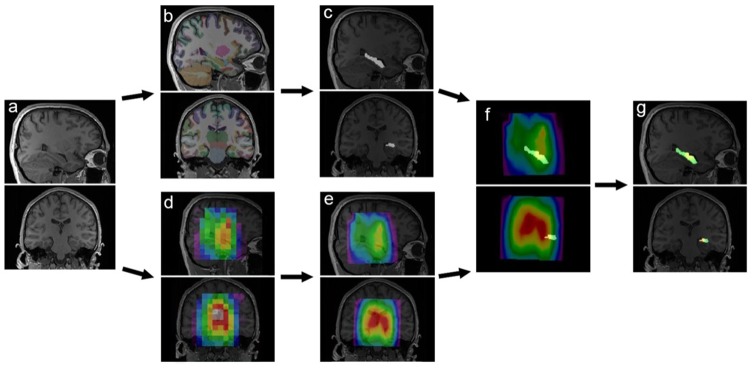
Illustration of automated region of interest (ROI)-specific magnetic resonance spectroscopy (MRS) analysis: structural T1-weighted MR images **(A)** are automatically segmented in cortical and subcortical areas using FreeSurfer **(B)**. Masks of individual ROIs are extracted **(C)**. Multi-voxel MRS data is resampled to the resolution of the MR images **(D,E)** and coregistered with individual masks **(F)**, resulting in distributions within single ROIs **(G)**.

3D-T_1_-weighted structural images of each individual scan were automatically segmented using FreeSurfer 6.0 in cortical and subcortical regions (Fischl et al., 1999, 2002; Desikan et al., 2006; Destrieux et al., 2010). In-house MATLAB codes were used for mask extraction of individual ROIs. GABA+/tNAA, Glx/tNAA and tNAA/tCr maps were interpolated to the resolution of the MPRAGE images (288 × 288 × 208) using nearest-neighbor interpolation and were overlaid with masks for each ROI (see Figure 1). An internal threshold for each ROI of 100% valid voxels per ROI for further quantification was set. ROIs which did not match quality criteria were excluded from further analysis. Mean GABA+/tNAA, Glx/tNAA, tNAA/tCr ratios and CRLB values were derived for individual ROIs.

### Comparison of Labeling Approaches

**Figure 2:**
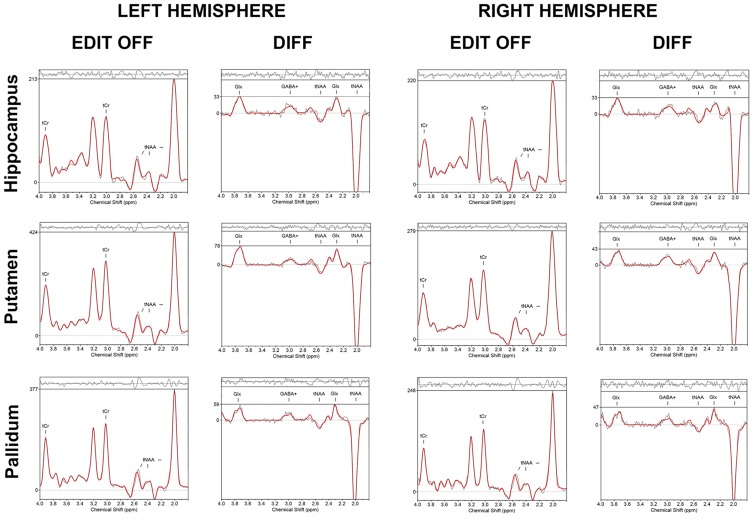
Exemplary *in vivo* proton MR spectra obtained with the gamma-aminobutyric acid (GABA)-editing MEGA-LASER 3D MRSI sequence from selected voxels of each ROI. The LCModel fit of metabolites in the EDIT-OFF and DIFF (difference spectrum; subtraction of EDIT-ON and EDIT-OFF) spectrum is shown, respectively.

For purposes of quality control, mean GABA+/tNAA and Glx/tNAA ratios from six regions (hippocampus, putamen and pallidum bilaterally) were compared between the automated labeling approach and manually drawn ROIs by two trained neuroscientists (rater 1 and rater 2) using MINC. Therefore, MRS data of 18 healthy subjects [10 female, mean age and standard deviation (25 ± 3 years) with no history of psychiatric disorders, neurodegenerative diseases or brain injuries] was used. Apart from the mask extraction, the same procedure as described for automated labeling was used in manual labeling for quality control and calculation of GABA+/tNAA and Glx/tNAA ratios. Moreover, a comparison between automated labeling and single voxel selection from a multi-voxel grid was performed. For this purpose, one selected voxel, within each desired region was chosen manually by one rater from the original grid (1 cm^3^ voxel size). Mean GABA+/tNAA and Glx/tNAA ratios derived from each voxel were compared to values derived from the automated labeling approach. Exemplary MRS-spectra of selected voxels are shown in Figure 2.

To validate automated labeling for different voxel sizes, spectral maps of eight healthy subjects [four female, mean age and standard deviation (23 ± 2 years)] were acquired for four regions (putamen and pallidum bilaterally) using non-edited high-resolution MRSI (0.65 cm^3^ voxel size). The same labeling procedure as described above was conducted with derived tNAA/tCr ratio maps.

### Statistical Analysis

To detect possible differences between the analysis methods, paired *t*-tests were performed using IBM SPSS Statistics (v25.0, 2010, SPSS, Inc., an IBM Company, Chicago, IL, USA). Overlay indices for each ROI were calculated between masks derived from automated labeling and each rater, as well as among both raters, using Szymkiewicz–Simpson coefficient. For consistency of mean ROI values of GABA+/tNAA, Glx/tNAA and tNAA/tCr ratios between automated labeling, manual labeling and selected voxels, intra-class correlation coefficients (ICC) were calculated using a two-way mixed model with absolute agreement, where values near 1 refer to absolute and 0 to no agreement between two measures (Weir, 2005). Furthermore, Bland-Altman analysis was conducted using MATLAB.

## Results

Szymkiewicz–Simpson coefficient revealed values >0.7 for each ROI and labeling comparison.

### Low-Resolution GABA-Edited MRS

**Figure 3:**
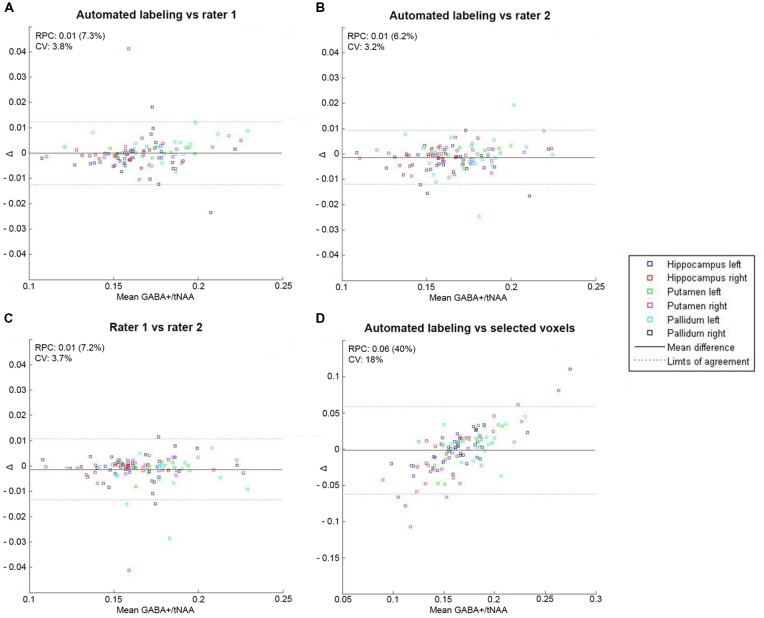
Bland-Altman plot with limits of agreement indicating 1.96*SD (dotted lines) for mean GABA+/total N-acetylaspartate (tNAA) ratios within all regions showing the agreement between two labeling methods for automated labeling vs. rater 1 **(A)**, automated labeling vs. rater 2 **(B)**, rater 1 vs. rater 2 **(C)** and automated labeling vs. selected voxels **(D)**. RPC, reproducibility coefficient and % of values; CV, coefficient of variation (SD of mean values in %).

**Figure 4:**
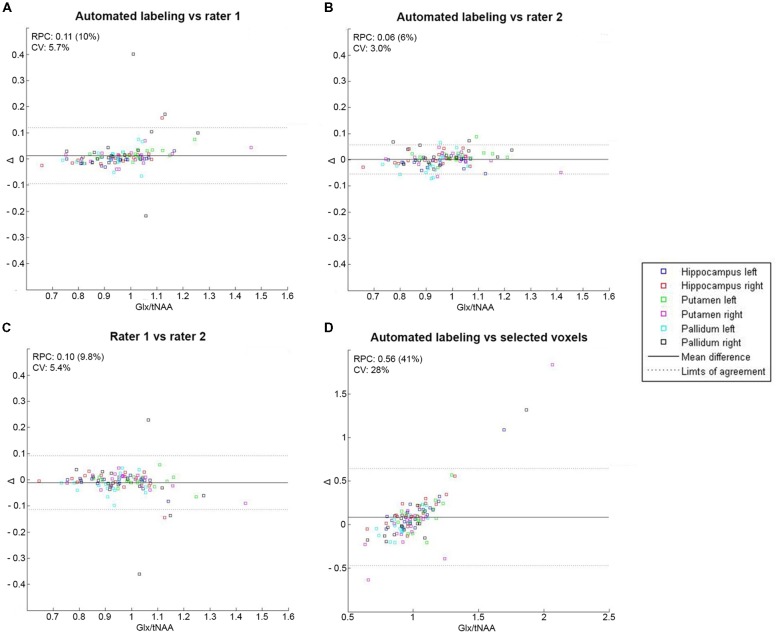
Bland-Altman plot with limits of agreement indicating 1.96*SD (dotted lines) for mean Glx/tNAA ratios within all regions showing the agreement between two labeling methods for automated labeling vs. rater 1 **(A)**, automated labeling vs. rater 2 **(B)**, rater 1 vs. rater 2 **(C)** and automated labeling vs. selected voxels **(D)**. RPC, reproducibility coefficient and % of values; CV, coefficient of variation (SD of mean values in %).

Mean GABA+/tNAA and Glx/tNAA showed no significant difference between automated/manual labeling and automated labeling/selected voxels in any region (*p* > 0.2). Data distribution and underlying CRLB values are displayed in Table 1. Bland-Altman plots showed high consistency within automated and manual labeling for each region [reproducibility coefficient (RPC) ≤0.01, or ≤8% of values for GABA+/tNAA, and RPC ≤ 0.11 or ≤10% of values for Glx/tNAA ratios], and low consistency between automated labeling and selected voxels [GABA+/tNAA: RPC = 0.06 (40%); Glx/tNAA: RPC = 0.56 (41%); see Figures 3, 4].

**Table 1:** Metabolite ratio mean, standard deviation (Stdev) and cramér-Rao lower bounds (CRLBs) values for each region and labeling method.

ROI		Automated labeling	Rater 1	Rater 2	Voxel
Hippocampus Left	Mean ± Stdev GABA+/tNAA	0.15 ± 0.01	0.15 ± 0.01	0.15 ± 0.01	0.15 ± 0.03
	CRLB GABA+	15.04	15.18	15.19	15.81
	Mean ± Stdev Glx/tNAA	0.96 ± 0.11	0.96 ± 0.11	0.95 ± 0.10	1.13 ± 0.32
	CRLB Glx	7.66	7.48	7.37	7.37
Hippocampus Right	Mean ± Stdev GABA+/tNAA	0.16 ± 0.02	0.16 ± 0.02	0.16 ± 0.02	0.15 ± 0.05
	CRLB GABA+	15.27	15.08	15.15	18.02
	Mean ± Stdev Glx/tNAA	0.93 ± 0.11	0.94 ± 0.13	0.93 ± 0.12	1.08 ± 0.23
	CRLB Glx	8.62	8.47	8.44	9.06
Putamen Left	Mean ± Stdev GABA+/tNAA	0.17 ± 0.02	0.17 ± 0.02	0.17 ± 0.02	0.18 ± 0.03
	CRLB GABA+	12.45	12.39	12.49	11.58
	Mean ± Stdev Glx/tNAA	1.01 ± 0.09	1.03 ± 0.10	1.02 ± 0.10	1.11 ± 0.18
	CRLB Glx	7.30	7.36	7.46	6.03
	Mean ± Stdev tNAA/tCr	1.28 ± 0.11	1.25 ± 0.12	1.23 ± 0.11	1.42 ± 0.53
Putamen Right	Mean ± Stdev GABA+/tNAA	0.17 ± 0.02	0.17 ± 0.02	0.17 ± 0.02	0.15 ± 0.06
	CRLB GABA+	13.53	13.93	13.84	17.82
	Mean ± Stdev Glx/tNAA	0.99 ± 0.14	1.00 ± 0.15	0.99 ± 0.13	1.03 ± 0.53
	CRLB Glx	9.62	9.88	10.05	11.67
	Mean ± Stdev tNAA/tCr	1.27 ± 0.25	1.23 ± 0.28	1.22 ± 0.28	1.18 ± 0.39
Pallidum Left	Mean ± Stdev GABA+/tNAA	0.18 ± 0.02	0.18 ± 0.02	0.18 ± 0.02	0.18 ± 0.03
	CRLB GABA+	11.78	11.64	11.95	11.46
	Mean ± Stdev Glx/tNAA	0.93 ± 0.08	0.93 ± 0.09	0.91 ± 0.09	0.92 ± 0.17
	CRLB Glx	7.61	7.84	8.73	7.91
	Mean ± Stdev tNAA/tCr	1.23 ± 0.19	1.26 ± 0.10	1.27 ± 0.10	1.35 ± 0.21
Pallidum Right	Mean ± Stdev GABA+/tNAA	0.17 ± 0.02	0.17 ± 0.02	0.17 ± 0.02	0.18 ± 0.05
	CRLB GABA+	14.07	14.50	14.26	13.89
	Mean ± Stdev Glx/tNAA	0.94 ± 0.12	0.98 ± 0.15	0.96 ± 0.12	1.00 ± 0.41
	CRLB Glx	9.14	9.39	9.33	9.54
	Mean ± Stdev tNAA/tCr	1.40 ± 0.15	1.38 ± 0.11	1.39 ± 0.15	1.64 ± 0.37

**Figure 5:**
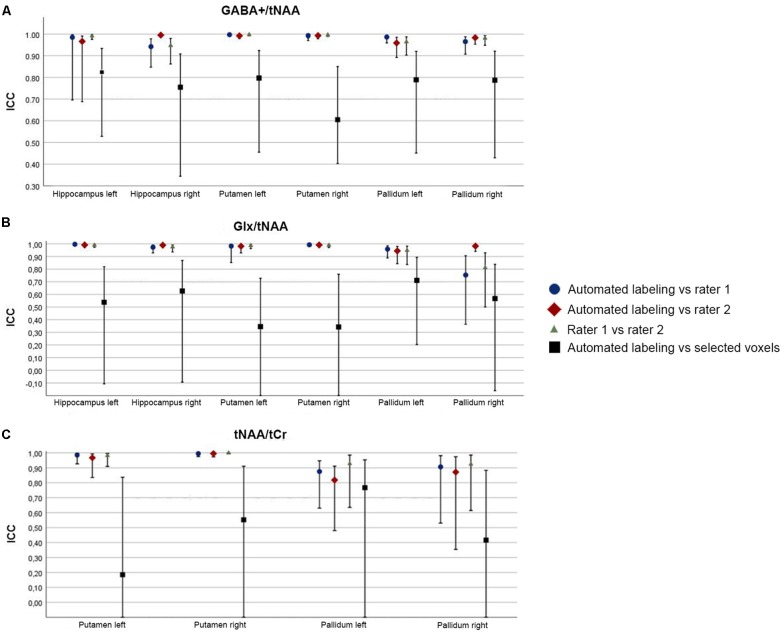
Graphical illustration of intra-class correlation coefficient (ICC) values for each region and metabolite ratio [ICC with upper and lower bound (error bars)] between labeling methods for GABA+/tNAA **(A)**, Glx/tNAA **(B)** and tNAA/tCr **(C)**.

ICC analysis revealed high consistency between automated labeling and each rater for manual labeling (ICC > 0.9), with highest deviation in the pallidum, see Figures 5A,B. ICC comparison between selected voxels and automated labeling showed lower consistency (ICC ranging from 0.35 to 0.83).

### High-Resolution Non-edited MRS

Mean tNAA/tCr showed no significant difference between automated/manual labeling and automated labeling/selected voxels in any region (*p* > 0.1). Bland-Altman analysis showed high consistency within automated and manual labeling for each region (RPC ≤0.2 or ≤15%), and low consistency between automated labeling and selected voxels [RPC = 0.73 (51%)]. ICC analysis revealed—similar to low-resolution data—a high consistency between automated labeling and each rater for manual labeling (ICC > 0.8), see Figure 5C. Poor consistency could be shown when selected voxels were compared with automated labeling (ICC ranging from 0.18 to 0.77).

## Discussion

This study aims to introduce an automated ROI-based labeling for multi-voxel MRS data. Previous studies relied on manual labeling approaches for ROIs or selected single voxels from a grid within a selected a region. Our method provides an unbiased approach for performing ROI-based analysis of multi-voxel MRS data using spatial averaging based on anatomical features. Furthermore, correction of underlying tissue types is automatically applied, depending on the ROI. Profound data analysis methods are of particularly high importance for metabolites, which differ in concentration according to the underlying tissue type (Jensen et al., 2005; Bhattacharyya et al., 2011; Harris et al., 2015). Regional metabolite ratios, calculated by ROI-based labeling, showed similar distributions compared to other studies (Bednařík et al., 2015). When analysis approaches were compared, automated ROI-based labeling showed solid results compared to manual ROI-based labeling and lower consistency with selected voxels from a multi-voxel grid in both low- and high-resolution MRS data (see Figure 5).

Metabolite ratios of selected voxels showed higher deviation within the group for all tested regions and lower consistency regarding clustering methods for ROI-based approaches. Smaller voxel sizes showed even worse results, when selected voxels were compared to automated or manual labeling. One can assume, that these effects result from the fact that voxel composition is neglected in selected voxels, whereas automated and manual labeling allow for better selection of GM-rich regions (Lai et al., 2018). Moreover, averaging across several voxels reduced error rates. Furthermore, Bland-Altman plots revealed systematic errors in extremes of the data distribution for selected voxels (see Figures 3D, 4D). Hence, it is crucial to include voxel composition (Porges et al., 2017) and neighboring voxels into the data analysis.

In the comparison of automated and manual labeling, similar distributions could be shown across different regions. Lowest variation was shown in the putamen, a visually definite region. However, in regions that are less visually defined, e.g., the pallidum, higher inter-rater variability could be detected. This in turn shows the importance of automated, unbiased labeling for MRS data.

The proposed automated analysis approach aims to be applicable for a broad range of 3D multi-voxel MRS analyses, independently of the acquisition method. However, the use is limited by the applicability and accuracy of automated cortical and subcortical segmentation of structural images. FreeSurfer has shown solid results in a sample of elderly patients (Liem et al., 2015) or when lesions are detected (Guo et al., 2018) which allows the use of this method in a clinical setting.

### Limitations

The signal contributions of adjacent voxels in a multi-voxel grid are a challenge for data analysis of multi-voxel MRS data. The signal derived from a selected region/voxel is always contaminated (Bradley et al., 2016). However, normalization of ROIs within subjects in a longitudinal study design helps to keep partial volume effects on a constant level. Furthermore, one has to consider that the proposed automated labeling approach can be applied if either the originally derived multi-voxel grid provides a sufficient resolution (Goryawala et al., 2016) or whenever high SNR allows downsampling of bigger voxel sizes. However, downsampling should be handled with caution since insufficient SNR within a region will increase error rates.

### Conclusion

This method provides a helpful tool for automated multi-voxel data analysis for the assessment of one or multiple ROIs. Especially, data analysis for longitudinal studies will benefit from using this approach, since metabolite concentrations can be derived in each region, regardless of exact voxel position during data acquisition. This approach yields several advantages compared to other analysis methods for multi-voxel MRS data. Automated ROI-based labeling enables MRS data analysis of desired regions applicable for a variety of different input data, with tremendously reduced hands-on time compared to automated labeling. Due to a masking method in the individual space inherent to FreeSurfer, a correction for changes in GM volume, e.g., due to atrophy in elderly patients, is applied. Hence, as a result of downsampling or clustering of the MRS data in desired regions, data can be disposed solely in gray or WM areas. In turn, correction models for voxel composition are not required (Porges et al., 2017). However, an automated labeling approach is to favor over manual labeling in terms of inter-rater bias. Especially regions that are challenging to draw manually are expected to yield better results when drawn in an automated manner.

## Data Availability

The datasets generated for this study are available on request to the corresponding author.

## Author Contributions

BS was responsible for conducting MRI scans, manual labeling, statistical analysis and writing of the manuscript. EH was responsible for manual labeling. WB and PM were responsible for technical support and MRS sequence development. RS and MK conducted MRI scans. MS and TV were responsible for medical assistance. RL was the scientific supervisor and principal investigator of the trial.

## Conflict of Interest Statement

RL received travel grants and/or conference speaker honoraria from Shire, AstraZeneca, Lundbeck A/S, Dr. Willmar Schwabe GmbH, Orphan Pharmaceuticals GA, Janssen-Cilag Pharma GmbH, and Roche Austria GmbH. MS has received travel grants from Janssen, Eli Lilly, and AOP Orphan Pharamceuticals, speaker honoraria from Janssen, and workshop participation from Eli Lilly. TV received travel grants and compensation for workshop participation from Pfizer and Eli Lilly and speaker honorary from Shire. The remaining authors declare that the research was conducted in the absence of any commercial or financial relationships that could be construed as a potential conflict of interest.

## Abbreviations

CRLB: Cramér-Rao lower bounds
CSF: cerebrospinal fluid
FOV: field of view
GABA: gamma-Aminobutyric acid
Gln: glutamine
Glu: glutamate
Glx: combination of glutamate and glutamine
GM: gray matter
ICC: intra-class correlation coefficient
MPRAGE: magnetization-prepared rapid gradient-echo sequence
MRS: magnetic resonance spectroscopy
ROI: region of interest
RPC: reproducibility coefficient
tCr: total creatine
SNR: signal-to-noise ratio
tNAA: total N-acetylaspartate
VOI: volume of interest
WM: white matter.
